# Associations of Obesity With Growth and Puberty in Children: A Cross-Sectional Study in Fuzhou, China

**DOI:** 10.3389/ijph.2023.1605433

**Published:** 2023-05-15

**Authors:** Ying Zhang, Xin Yuan, XiaoHong Yang, XiangQuan Lin, ChunYan Cai, ShiJun Chen, ZhuanZhuan Ai, HuaKun ShangGuan, WenYong Wu, RuiMin Chen

**Affiliations:** Department of Endocrinology, Genetics and Metabolism, Fuzhou Children’s Hospital of Fujian Medical University, Fuzhou, China

**Keywords:** children, obesity, growth, precocious puberty, menarche

## Abstract

**Objectives:** To investigate the associations of obesity with growth and puberty in children.

**Methods:** From November 2017 to December 2019, height, weight, and Tanner stages of 26,879 children aged 3–18 years in Fuzhou, China were assessed.

**Results:** The obese group was significantly taller than the non-obese group after age 4 years for both genders, yet there was no significant difference in height between obese and non-obese group after 15.5 years old for boys and 12.5 years old for girls. The inflection points of significant growth deceleration in obese and non-obese groups were 14.4 and 14.6 years old for boys, and 11.8 and 12.8 years old for girls, respectively. The proportions of testicular development in boys with obesity and non-obesity were 7.96% and 5.08% at 8.5–8.9 years old, respectively, while the proportions of breast development in girls were 17.19% and 3.22% at age 7.5–7.9 years old, respectively.

**Conclusion:** Children with obesity were taller in early childhood, earlier onset of puberty and earlier cessation of growth than children with non-obesity of the same age. However, there was sex dimorphism on the effect of obesity on the incidence of precocious puberty.

## Introduction

Childhood obesity is a global health problem, with the prevalence of obesity in children at all ages increasing at an alarming rate over the past 40 years [1]. Obesity can be affected by many factors, such as genetic predisposition, intrauterine environment, socioeconomic status and health literacy. Incontrovertibly, ultra-processed foods and reduced physical activity play important roles [2, 3].

Mammalian growth is an intricate physiologic process, affected by nutrition, genes and hormones [4]. Insofar as nutrition plays an important regulatory role in growth and development, it follows that childhood obesity may impact onset and duration of puberty [5–7]. The association between obesity and growth is germane due to the increasing prevalence of childhood obesity worldwide [8]. For example, pubertal height gain is inversely related to peak BMI [5, 6].

The secular trend toward earlier childhood puberty onset has been observed in recent decades, possibly attributable to improvements in nutrition and healthcare [9–11]. Early puberty may be associated with an increased future risk of multiple diseases, such as cardiovascular disease, depression, type 2 diabetes and cancers, with clinical implications for treatment of children [12]. Epidemiological studies have demonstrated a link between earlier onset of puberty in girls with high BMI or obesity [13, 14], while this phenomenon remains inconsistent in boys. Some studies found a positive association between BMI and earlier onset of puberty [15], while others demonstrated delayed onset of puberty [16] or no significant relation in boys with obesity [17].

Frequently, children with obesity are taller for their age, with accelerated linear growth and advanced skeletal maturation, and tend to mature earlier than lean counterparts [18]. Although adequate nutrition is essential for normal growth in children with obesity are usually taller than their normal-weight peers, yet they do not tend to be taller height as adults [19]. A large Swedish study [6] found that puberty started earlier, and the height gain during puberty was reduced in children with obesity. This later reduction occurred despite an association of increased BMI during ages 2–8 years and with a parallel gain in height. Observational studies [19, 20] have corroborated an earlier pubertal onset and alternated linear growth during puberty in those with an elevated BMI.

The curvilinear relationship between obesity and height, and the effect of obesity on puberty, is unresolved, especially in boys. This study evaluated the associations of obesity with growth and puberty in Chinese children based on a large-scale epidemiological data base.

## Methods

### Participants and Sampling

Data was extracted from the Fuzhou component of PRODY study from November 2017 to December 2019 using a stratified cluster-sampling design. Kindergartens, primary and senior high schools were randomly sampled from lists in Fuzhou, Fujian, China. Children and adolescents from 34 kindergartens, 13 primary schools and 12 senior high schools were recruited. The questionnaires encompassed birth history, past history, diet and exercise habits, and were completed by parents. Children who had been diagnosed with organic disease (such as germ cell tumor, congenital heart disease, etc.), chronic diseases (such as liver and chronic kidney disease, etc.), genital abnormalities (such as hypospadias, cryptorchidism, etc.) were excluded from the study.

Given the detection rate of precocious puberty was 6.65% in Shanghai, China [21], or our estimated sample size was 5,615. The detection rate of obesity was 8.31% for boys and 4.12% for girls in Guangzhou, China [22], and our estimated sample size was 9,308. In total, 26,879 healthy children, including 14,647 boys and 12,232 girls, who completed the questionnaire and physical examination were included in the final analysis (Supplementary Figure S1).

### Anthropometric Measurement

Anthropometric measures while wearing light clothing, without shoes, were done by professional endocrinologists or pediatricians. Height and weight were measured with uniform and calibrated instruments. Waist circumference (WC) was measured at the midpoint of the horizontal line between the lower rib margin and the superior iliac border using an inelastic measuring tape. Hip circumference (HC) was measured at the maximum protuberance of the buttocks. The measurement of height, WC and HC were recorded with a precision of 0.1 cm, and weight was recorded with a precision of 0.5 kg.

The participants were defined as obese according to the sex-age-specific cut-off point standard of body mass index (BMI) for children and adolescents aged 0–18 years in China [23]. The BMI cut-off corresponded to a BMI of 28 kg/m^2^ (greater than the 96.3rd percentile in boys and greater than the 98th percentile in girls) at the age of 18 years (Supplementary Table S1).

### Sexual Character Examination

Sexual development, including testes, breast and pubic hair, were assessed by a pediatric endocrinologist. Testicular (T) volume for boys was determined by palpation and volume determined by orchidometer. Breast (B) development in girls was evaluated by inspection combined with palpation. Pubic hair (PH) development in both genders were evaluated on exam. The age of menarche was obtained by asking the girls themselves during the physical examination, which were divided into different age groups: none, <10 years, 10–11 years, 11–12 years, 12–13 years, 13–14 years, 14–15 years and >15 years. The pubertal stage was assessed by standard Tanner staging [24, 25]. Precocious puberty was defined as secondary sexual characteristics before 9 years of age in boys and before 8 years of age in girls [26, 27].

### Statistical Analysis

The collected data were recorded and proofread by two groups of researchers. Basic characteristics of the participants, such as the number of participants with obesity and precocious puberty and the means (standard deviation, SD) of age, height and BMI by sex, were calculated. A probit regression analysis was used to estimate the median age of reaching or above T2, B2 and PH2. A generalized additive model (GAM) was used to identify the non-linear relationships. A two-piece wise linear regression model was constructed to calculate the threshold effect of age on height status in terms of a smoothing plot if a non-linear relationship was observed. When the relationship between age status and height levels is conspicuous in this smoothed curve, the recursive method automatically calculated the inflection point, where the maximum model likelihood would be used [28]. Continuous and categorical variables were compared between groups using t-tests or chi-square tests, respectively, and a *p* value <0.05 was considered statistically significant. R software (http://www.R-project.org) and Empower (R) (www.empowerstats.com; X&Y Solutions, Inc., Boston, MA, United States) were used for all statistical analyses.

## Results

### Characteristics of Subjects

**FIGURE 1:**
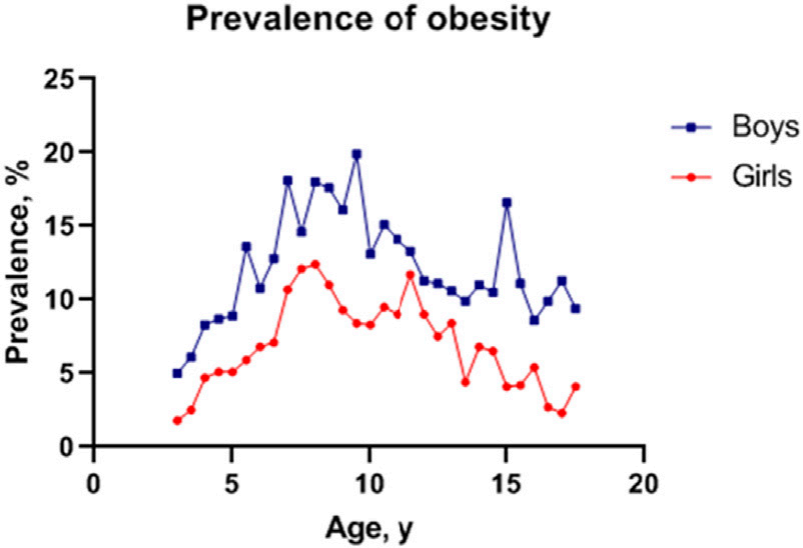
Sex- and age-specific prevalence of obesity in Fuzhou, China, 2017–2019.

A total of 26,879 participants, including 14,647 boys and 12,232 girls, were included in the final analysis (Table 1). Boys scored significantly higher than girls in height, BMI, WC and HC (*p* < 0.01). In general, the obesity prevalence in boys was higher than girls across the age group, with a prevalence of obesity of 10.42% (12.85% for boys and 7.50% for girls) aged 3–18 years (Figure 1).

**TABLE 1:** Basic characteristics of the subjects (China, 2017–2019).

	Boys	Girls	*t* value	*p* value
n	14,647	12,232		
Obesity(n)	1,882	918		
Age (years)	10.08 ± 3.78	10.00 ± 3.83	1.702	0.089
Height (cm)	140.95 ± 22.11	137.45 ± 19.77	13.553	<0.001
BMI	18.65 ± 3.87	17.68 ± 3.38	21.608	<0.001
Waist (cm)	64.36 ± 12.30	59.39 ± 10.36	35.443	<0.001
Hip (cm)	72.54 ± 13.76	71.28 ± 13.17	7.641	<0.001
Waist/Hip	0.89 ± 0.10	0.84 ± 0.11	39.621	<0.001

### Associations of Obesity With Growth

**FIGURE 2:**
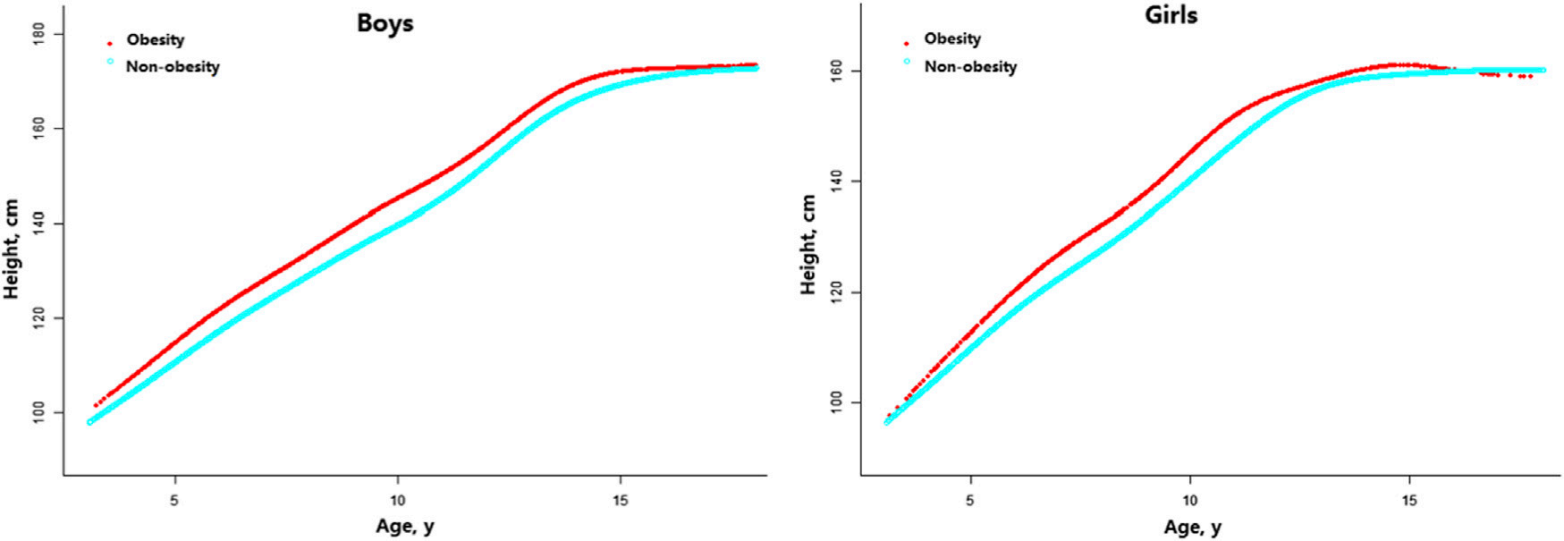
Age-specific height differences between children with obesity and non-obesity (China, 2017–2019).

As per Figure 2, obesity had an obvious influence on growth in both genders. For boys, the obesity group was significantly taller than the non-obesity group after 4 years of age, yet the significant difference in height vanished after 15.5 years of age. The height gap in boys reached a maximum at the age of 9.5–9.9 years old, with a mean difference of 6.44 cm between the obesity and non-obesity groups. For girls, the obesity group was significantly taller than the non-obesity group after 4 years of age, and no significant difference in height was evident after 12.5 years of age. The height gap in girls reached a maximum at the age of 11.0–11.4 years old, with a mean difference of 5.74 cm between the obesity and non-obesity groups (Supplementary Tables S2, S3).

By using a two-piece linear regression model, a non-linear relationship was found between height and age. Results showed that the inflection point of significant growth deceleration was 14.6 and 14.4 years old in boys with obesity and non-obesity, respectively, while it was 12.8 and 11.8 years old in girls with obesity and non-obesity, respectively (Table 2).

**TABLE 2:** The results of two-piece wise linear regression model between height and age in children with obesity and non-obesity (China, 2017–2019).

	Group	Inflection points of age (years)	Left of inflection point (cm) (β, 95% CI, *p* value)	Right of inflection point (cm) (β, 95% CI, *p* value)	Equation predictions at inflection point (cm) (β, 95% CI)	*p* value
Boys	Obesity	14.4	6.0 (6.0, 6.1)	0.3 (−0.1, 0.8)	171.9 (171.3, 172.4)	<0.001
			<0.001	0.170		
	Non-obesity	14.6	6.1 (6.0, 6.1)	1.4 (1.2, 1.6)	168.8 (168.6, 169.0)	<0.001
			<0.001	<0.001		
	Total	14.4	6.1 (6.0, 6.1)	1.2 (1.1, 1.4)	169.4 (169.2, 169.6)	<0.001
			<0.001	<0.001		
Girls	Obesity	11.8	6.5 (6.4, 6.7)	0.9 (0.5, 1.2)	157.0 (156.3, 157.7)	<0.001
			<0.001	<0.001		
	Non-obesity	12.8	6.2 (6.1, 6.2)	0.6 (0.6, 0.7)	157.8 (157.6, 158.0)	<0.001
			<0.001	<0.001		
	Total	12.7	6.2 (6.2, 6.2)	0.7 (0.6, 0.8)	157.7 (157.5, 157.9)	<0.001
			<0.001	<0.001		

### Associations of Obesity With Pubertal Stages

A probit analysis was used to estimate differences in median age at various Tanner stages (Table 3). For boys, the median age of T2 was 10.90 and 11.38 years in the obesity and non-obesity groups, respectively, and the median age of PH2 was 12.15 and 12.59 years in the obesity and non-obesity groups, respectively. For girls, the median age of B2 was 9.18 and 9.82 years in the obesity and non-obesity cohort, respectively, and the median age of PH2 was 10.43 and 11.28 years in the obesity and non-obesity groups, respectively. Compared with the non-obesity group, the median age of Tanner 2 in the obesity group was significantly earlier in both boys and girls (*p* < 0.05).

**TABLE 3:** Age and height at different pubertal stages according to probit analysis (China, 2017–2019).

	Pubertal stages	n		Age (years)		t value	*p* value	Height (cm)		*t* value	*p* value
		Obesity	Non-obesity	Obesity	Non-obesity			Obesity	Non-obesity		
Boys	T2	270	1,350	10.90 (10.07–11.70)	11.38 (10.64–12.16)	6.591	<0.001	149.30 ± 9.28	146.94 ± 8.38	4.018	<0.001
	T3	142	1,328	13.18 (12.22–14.28)	13.38 (12.49–14.37)	1.289	0.198	163.81 ± 8.66	161.41 ± 8.47	3.207	0.001
	T4	191	1,602	14.55 (13.45–15.87)	14.52 (13.52–16.08)	0.989	0.323	169.41 ± 6.99	167.93 ± 7.13	2.711	0.007
	T5	186	1,217	15.12 (13.86–16.29)	15.38 (14.13–16.60)	2.533	0.011	171.92 ± 6.53	170.64 ± 6.46	2.516	0.012
	PH2	63	472	12.15 (11.29–12.89)	12.59 (11.68–13.26)	2.590	0.010	156.85 ± 8.68	154.50 ± 7.85	1.857	0.064
	PH3	84	912	12.90 (12.18–13.55)	13.28 (12.69–13.87)	3.192	0.001	163.30 ± 6.24	162.28 ± 7.63	1.149	0.251
	PH4	80	795	13.67 (13.14–14.15)	13.94 (13.30–14.47)	1.849	0.065	168.52 ± 6.07	167.06 ± 6.56	1.912	0.056
	PH5	306	2,010	15.38 (14.36–16.45)	15.92 (14.80–16.72)	4.361	<0.001	172.34 ± 6.10	170.91 ± 6.49	3.749	<0.001
Girls	B2	85	948	9.18 (8.32–9.90)	9.82 (8.98–10.47)	4.863	<0.001	138.37 ± 7.39	137.96 ± 7.56	0.478	0.633
	B3	115	1,088	9.99 (9.03–10.78)	11.15 (10.39–12.27)	10.017	<0.001	145.26 ± 7.85	148.87 ± 7.77	4.735	<0.001
	B4	92	1,516	11.35 (10.81–12.35)	13.21 (12.20–14.19)	9.938	<0.001	154.28 ± 6.05	156.75 ± 6.22	3.713	<0.001
	B5	232	2,346	13.77 (12.87–15.14)	15.28 (13.93–16.34)	10.785	<0.001	159.05 ± 6.10	159.28 ± 5.56	0.613	0.540
	PH2	39	505	10.43 (10.02–11.18)	11.28 (10.63–12.10)	3.709	<0.001	150.25 ± 7.02	149.05 ± 7.75	0.933	0.351
	PH3	61	719	11.47 (10.99–12.13)	12.62 (11.64–13.51)	5.205	<0.001	154.64 ± 6.92	155.21 ± 5.91	0.707	0.480
	PH4	81	1,243	12.87 (11.90–13.79)	13.76 (12.94–14.52)	6.020	<0.001	156.86 ± 6.05	158.22 ± 5.74	2.186	0.029
	PH5	167	2011	14.13 (13.12–15.73)	15.54 (14.21–16.44)	8.537	<0.001	160.01 ± 5.55	159.65 ± 5.41	0.810	0.418

**FIGURE 3:**
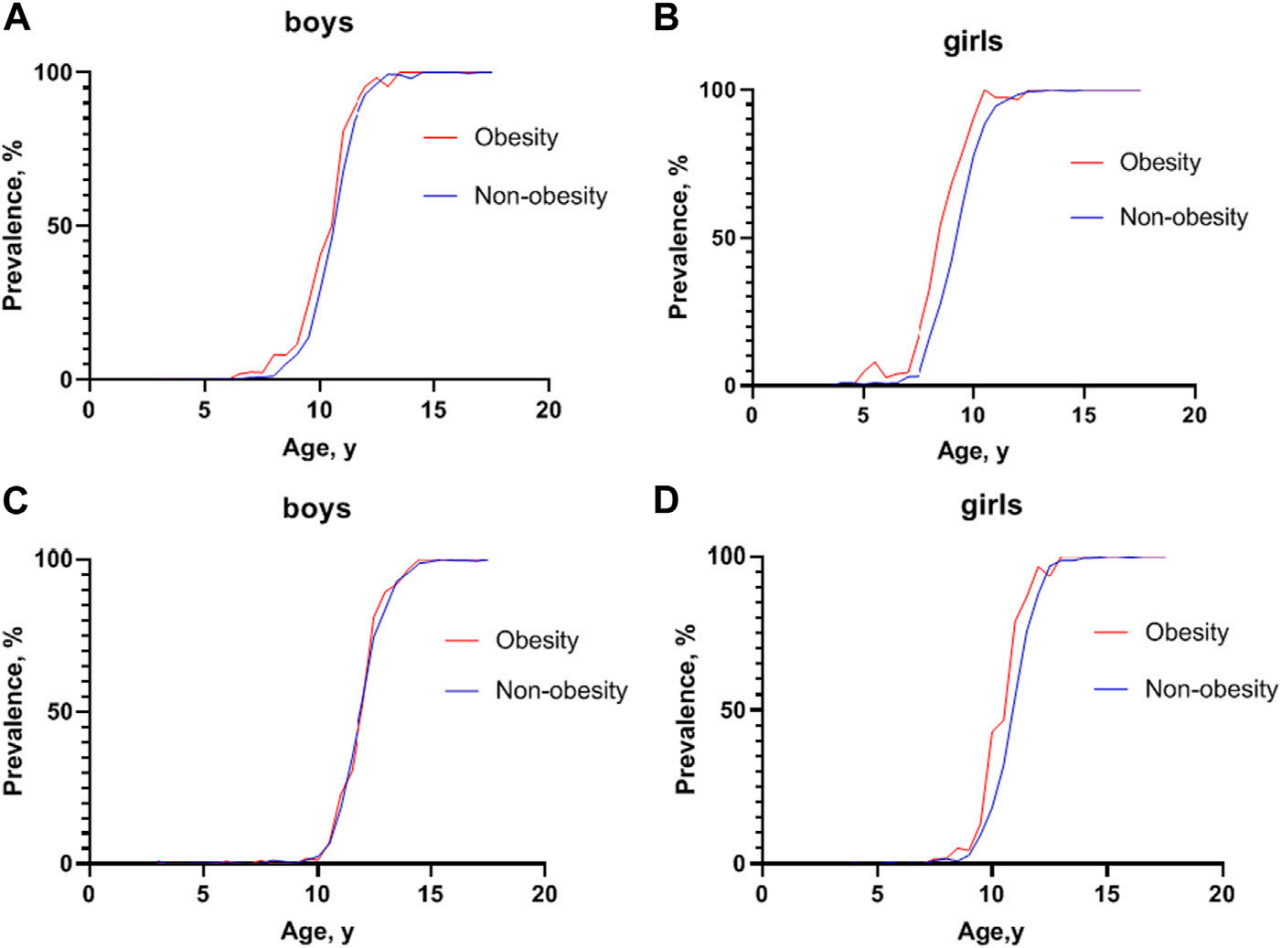
Prevalence of sexual development at different ages between children with obesity and non-obesity (China, 2017–2019). **(A)** Prevalence of testicular development at Tanner stage 2 or greater at different ages in boys; **(B)** Prevalence of breast development at Tanner stage 2 or greater at different ages in girls; **(C)** Prevalence of pubic hair development at Tanner stage 2 or greater at different ages in boys; **(D)** Prevalence of pubic hair development at Tanner stage 2 or greater at different ages in girls.

However, a sex dimorphism in the effect of obesity on the incidence of precocious puberty was observed. The proportions of testicular development in boys with obesity and non-obesity were 7.96% and 5.08% at 8.5–8.9 years old (*χ* = 1.287, *p* > 0.05), respectively, while the proportions of breast development in girls with obesity and non-obesity were 17.19% and 3.22% at age 7.5–7.9 years old (*χ* = 19.411, *p* < 0.001), respectively (Figure 3 and Supplementary Tables S4, S5).

Furthermore, a gender difference was noted on the effect of obesity on pubic hair development. The rates of pubic hair development in boys with obesity and non-obesity across age groups were similar, while girls with obesity developed pubic hair earlier than girls with non-obesity (Figure 3 and Supplementary Tables S6, S7).

Menarche in the girls with obesity was younger than that in the girls with non-obesity: 3,772 girls with non-obesity and 277 girls with obesity had menarche, and the proportions of menarche that occurred in less than 10 and 10–11 years were 3.25% and 23.10% in girls with obesity, respectively, compared with 1.14% and 11.98% in girls with non-obesity (Supplementary Table S8).

## Discussion

This study assessed the relationship between obesity and linear growth and pubertal development (as measured by breast, menarche, pubic hair and testes) based on a large-scale, population-based, cross-sectional survey in Fuzhou, China. The overall rate of obesity was 10.42% (12.85% for boys and 7.50% for girls) in the present study, which is higher than the previously reported 8.9% in China [29]. Our study found different growth curves in children with obesity and a positive relationship between obesity and puberty onset.

Herein, we found that children with obesity were taller than children with non-obesity after the age of 4 years, while this difference waned during puberty, resulting in no advantage in late adolescence. Our results established specific age and growth inflection points. In previous studies, children with obesity grew faster during prepubertal years but lost their growth advantage at puberty, ultimately resulting in no positive effect on adult height [5, 6, 30–32]. Advanced bone maturation due to obesity may explain the attenuated growth during puberty [32, 33]. In addition, we found that children with obesity ceased growing earlier, and this disparity was more noticeable in girls.

A secular trend toward an earlier onset of puberty has been observed in recent years for both girls and boys [34, 35]. Extensive studies have found a positive association between obesity (high BMI) and earlier puberty in girls, which has been confirmed by population-based cross-sectional and longitudinal studies in many diverse countries [12, 36–38]. Our results are consistent with most studies. Furthermore, a decrease in the median age of both thelarche and pubarche in girls with obesity were observed, and the age of menarche differed between non-obese and obese girls.

Unlike girls, the relationship between obesity and early puberty remains controversial in boys. Some studies have reported later puberty onset in boys with obesity. A large cross-sectional study involving 1,500 girls and 1,520 boys in the US found that early sexual maturation was reversely associated with overweight and obesity in boys [39]. However, other studies reacted antithetical conclusions. A large cohort study of Danish boys with voice breaks, considered as the marker of puberty, found a significant positive association between higher BMI and earlier puberty [40]. In a cross-sectional study in Shanghai, China, a positive association between early puberty and obesity was observed, as evidenced by an advance in testicular development with increasing BMI [21]. The lack of consistent evidence for the effect of weight status on the timing of puberty in boys may be due to differences in ethnic background, sample size, method for puberty staging and statistical approach. In our study, boys in the obesity group had a younger median age at T2 and PH2 than those in the non-obesity group. Nonetheless, the rates of precocious puberty in boys with obesity and non-obesity across age groups were similar. Puberty onset dimorphism existed between genders: although boys with obesity developed puberty earlier than boys with non-obesity on the whole, the effects of obesity were more apparent in girls than in boys.

Several potential mechanisms could explain the link between childhood obesity and early pubertal onset. Obesity has been proposed as a activated metabolic gatekeeper of puberty, one of the key factors mediating this effect of obesity on puberty may be related to adipokines, especially leptin [41], which can directly stimulate gonadotropin secretion. In addition, the association between childhood obesity and early puberty may be related to the status of hyperinsulinemia/insulin resistance associated with obesity and the genetic polymorphism of obesity [42]. The reason for sexual dimorphism is unknown, but plausibly the difference may be related to sex specificity actions of leptin [43, 44] and the interaction of kisspeptin with gonadotropin-releasing hormone [44, 45]. Kisspeptin and kisspeptin receptors in the hypothalamus, essential for the onset of puberty in both males and females, show a high degree of sexual dimorphism in expression and function and induce different GnRH secretion patterns in males and females [46].

### Strengths and Limitations of This Study

This study had several strengths, including the use of multiple pubertal evaluation indicators, deriving growth curves, establishing growth inflection points, and confirming the relationship between obesity and early puberty. The population was exclusive Chinese from Fuzhou, hence, diet, lifestyles, and health tend to be homogeneous.

However, there are limitations. First, the cross-sectional design cannot determine the causal relationship of puberty and obesity, nor the speed of the consecutive pubertal stages, and accurately determine the precise onset of puberty. Second, the study did not assess bone age, hence, it is infeasible to determine whether changes in bone age were associated with the effects of obesity on height. Furthermore, there may be some inaccuracies in breast palpation in girls with obesity, given the challenge to distinguish adipose vs. breast tissue, and there may be recall error as to the age of menarche.

### Conclusion

In this cross-sectional study, differences in growth curves and growth inflection points between children with obesity and non-obesity were found, corroborating that children with obesity were taller in early childhood, earlier onset of puberty and earlier cessation of growth than children with non-obesity of the same age, but there was sex dimorphism in the effect of obesity on the incidence of precocious puberty. In sum, these findings underscore the importance of assessing physical development and the onset and progression of puberty in children with obesity, and provide guidance to healthcare providers as to when a referral to endocrinology is necessary.

## References

[1] ANCD Risk Factor Collaboration (NCD-RisC). Worldwide Trends in Body-Mass index, Underweight, Overweight, and Obesity from 1975 to 2016: a Pooled Analysis of 2416 Population-Based Measurement Studies in 128.9 Million Children, Adolescents, and Adults. Lancet (2017) 390(10113):2627–42. 10.1016/S0140-6736(17)32129-3 29029897 PMC5735219

[2] Elizabeth L Machado P Zinöcker M Baker P Lawrence M. Ultra-Processed Foods and Health Outcomes: A Narrative Review. Nutrients (2020) 12(7):1955. 10.3390/nu12071955 32630022 PMC7399967

[3] Ten Velde G Plasqui G Dorenbos E Winkens B Vreugdenhil A. Objectively Measured Physical Activity and Sedentary Time in Children with Overweight, Obesity and Morbid Obesity: a Cross-Sectional Analysis. BMC Public Health (2021) 21(1):1558. 10.1186/s12889-021-11555-5 34404361 PMC8369633

[4] Marcovecchio ML Chiarelli F. Obesity and Growth during Childhood and Puberty. World Rev Nutr Diet (2013) 106:135–41. 10.1159/000342545 23428692

[5] Holmgren A Martos-Moreno GÁ Niklasson A Martínez-Villanueva J Argente J Albertsson-Wikland K. The Pubertal Growth Spurt Is Diminished in Children with Severe Obesity. Pediatr Res (2021) 90(1):184–90. 10.1038/s41390-020-01234-3 33173182

[6] Holmgren A Niklasson A Nierop AF Gelander L Aronson AS Sjöberg A Pubertal Height Gain Is Inversely Related to Peak BMI in Childhood. Pediatr Res (2017) 81(3):448–54. 10.1038/pr.2016.253 27861464

[7] Brix N Ernst A Lauridsen LLB Parner ET Arah OA Olsen J Childhood Overweight and Obesity and Timing of Puberty in Boys and Girls: Cohort and Sibling-Matched Analyses. Int J Epidemiol (2021) 49(3):834–44. 10.1093/ije/dyaa056 PMC7394964 32372073

[8] Costa de Miranda R Di Renzo L Cupertino V Romano L De Lorenzo A Salimei C Secular Trend of Childhood Nutritional Status in Calabria (Italy) and the United States: the Spread of Obesity. Nutr Res (2019) 62:23–31. 10.1016/j.nutres.2018.10.008 30803504

[9] Eckert-Lind C Busch AS Petersen JH Biro FM Butler G Bräuner EV Worldwide Secular Trends in Age at Pubertal Onset Assessed by Breast Development Among Girls: A Systematic Review and Meta-Analysis. JAMA Pediatr (2020) 174(4):e195881. 10.1001/jamapediatrics.2019.5881 32040143 PMC7042934

[10] Bräuner EV Busch AS Eckert-Lind C Koch T Hickey M Juul A. Trends in the Incidence of Central Precocious Puberty and Normal Variant Puberty Among Children in Denmark, 1998 to 2017. JAMA Netw Open (2020) 3(10):e2015665. 10.1001/jamanetworkopen.2020.15665 33044548 PMC7550972

[11] Sun Y Tao FB Su PY Mai JC Shi HJ Han YT National Estimates of the Pubertal Milestones Among Urban and Rural Chinese Girls. J Adolesc Health (2012) 51(3):279–84. 10.1016/j.jadohealth.2011.12.019 22921139

[12] Day FR Elks CE Murray A Ong KK Perry JR. Puberty Timing Associated with Diabetes, Cardiovascular Disease and Also Diverse Health Outcomes in Men and Women: the UK Biobank Study. Sci Rep (2015) 5:11208. 10.1038/srep11208 26084728 PMC4471670

[13] Currie C Ahluwalia N Godeau E Nic Gabhainn S Due P Currie DB. Is Obesity at Individual and National Level Associated with Lower Age at Menarche? Evidence from 34 Countries in the Health Behaviour in School-Aged Children Study. J Adolesc Health (2012) 50(6):621–6. 10.1016/j.jadohealth.2011.10.254 22626490

[14] Liu Y Yu T Li X Pan D Lai X Chen Y Prevalence of Precocious Puberty Among Chinese Children: a School Population-Based Study. Endocrine (2021) 72(2):573–81. 10.1007/s12020-021-02630-3 33528762

[15] Li Y Ma T Ma Y Gao D Chen L Chen M Adiposity Status, Trajectories, and Earlier Puberty Onset: Results from a Longitudinal Cohort Study. J Clin Endocrinol Metab (2022) 107(9):2462–72. 10.1210/clinem/dgac395 35779008

[16] Kleber M Schwarz A Reinehr T. Obesity in Children and Adolescents: Relationship to Growth, Pubarche, Menarche, and Voice Break. J Pediatr Endocrinol Metab (2011) 24(3-4):125–30. 10.1515/jpem.2011.089 21648278

[17] Abou El Ella SS Barseem NF Tawfik MA Ahmed AF. BMI Relationship to the Onset of Puberty: Assessment of Growth Parameters and Sexual Maturity Changes in Egyptian Children and Adolescents of Both Sexes. J Pediatr Endocrinol Metab (2020) 33(1):121–8. 10.1515/jpem-2019-0119 31851614

[18] Li W Liu Q Deng X Chen Y Liu S Story M. Association between Obesity and Puberty Timing: a Systematic Review and Meta-Analysis. Int J Environ Res Public Health (2017) 14(10):1266. 10.3390/ijerph14101266 29064384 PMC5664767

[19] Stovitz SD Demerath EW Hannan PJ Lytle LA Himes JH. Growing into Obesity: Patterns of Height Growth in Those Who Become normal Weight, Overweight, or Obese as Young Adults. Am J Hum Biol (2011) 23(5):635–41. 10.1002/ajhb.21191 21630370 PMC3152584

[20] Aris IM Rifas-Shiman SL Zhang X Yang S Switkowski K Fleisch AF Association of BMI with Linear Growth and Pubertal Development. Obesity (Silver Spring) (2019) 27(10):1661–70. 10.1002/oby.22592 31479205 PMC6756952

[21] Chen C Zhang Y Sun W Chen Y Jiang Y Song Y Investigating the Relationship between Precocious Puberty and Obesity: a Cross-Sectional Study in Shanghai, China. BMJ Open (2017) 7(4):e014004. 10.1136/bmjopen-2016-014004 PMC5566589 28400459

[22] Zong Y Xie R Deng N Liu L Tan W Gao Y Secular Trends in Overweight and Obesity Among Urban Children and Adolescents, 2003-2012: A Serial Cross-Sectional Study in Guangzhou, China. Sci Rep (2017) 7(1):12042. 10.1038/s41598-017-12094-z 28935860 PMC5608869

[23] Li H Ji C Zong X Zhang YQ. Body Mass index Growth Curves for Chinese Children and Adolescents Aged 0 to 18 Years. Zhonghua Er Ke Za Zhi (2009) 47(7):493–8. 19951508

[24] Marshall W Tanner J. Variations in Pattern of Pubertal Changes in Girls. Arch Dis Child (1969) 44(235):291–303. 10.1136/adc.44.235.291 5785179 PMC2020314

[25] Marshall W Tanner J. Variations in the Pattern of Pubertal Changes in Boys. Arch Dis Child (1970) 45(239):13–23. 10.1136/adc.45.239.13 5440182 PMC2020414

[26] Bridges NA Christopher JA Hindmarsh PC Brook CG. Sexual Precocity: Sex Incidence and Aetiology. Arch Dis Child (1994) 70(2):116–8. 10.1136/adc.70.2.116 8129431 PMC1029712

[27] Cheuiche AV da Silveira LG de Paula LCP Lucena IRS Silveiro SP. Diagnosis and Management of Precocious Sexual Maturation: an Updated Review. Eur J Pediatr (2021) 180(10):3073–87. 10.1007/s00431-021-04022-1 33745030

[28] Vittinghoff E McCulloch CE Glidden DV Shiboski SC. Linear and Non-linear Regression Methods in Epidemiology and Biostatistics. In: Epidemiology and Medical Statistics (2007). 148–86.

[29] Zhang L Chen J Zhang J Wu W Huang K Chen R Regional Disparities in Obesity Among a Heterogeneous Population of Chinese Children and Adolescents. JAMA Netw Open (2021) 4(10):e2131040. 10.1001/jamanetworkopen.2021.31040 34698846 PMC8548942

[30] Li Y Gao D Liu J Yang Z Wen B Chen L Prepubertal BMI, Pubertal Growth Patterns, and Long-Term BMI: Results from a Longitudinal Analysis in Chinese Children and Adolescents from 2005 to 2016. Eur J Clin Nutr (2022) 76:1432–9. 10.1038/s41430-022-01133-2 35523866

[31] Davison KK Susman EJ Birch LL. Percent Body Fat at Age 5 Predicts Earlier Pubertal Development Among Girls at Age 9. Pediatrics (2003) 111(4):815–21. 10.1542/peds.111.4.815 12671118 PMC2530923

[32] Denzer C Weibel A Muche R Karges B Sorgo W Wabitsch M. Pubertal Development in Obese Children and Adolescents. Int J Obes (Lond) (2007) 31(10):1509–19. 10.1038/sj.ijo.0803691 17653066

[33] Burt Solorzano CM McCartney CR. Obesity and the Pubertal Transition in Girls and Boys. Reproduction (2010) 140(3):399–410. 10.1530/REP-10-0119 20802107 PMC2931339

[34] Meng X Li S Duan W Sun Y Jia C. Secular Trend of Age at Menarche in Chinese Adolescents Born from 1973 to 2004. Pediatrics (2017) 140(2):e20170085. 10.1542/peds.2017-0085 28716824 PMC5527668

[35] Ohlsson C Bygdell M Celind J Sondén A Tidblad A Sävendahl L Secular Trends in Pubertal Growth Acceleration in Swedish Boys Born from 1947 to 1996. JAMA Pediatr (2019) 173(9):860–5. 10.1001/jamapediatrics.2019.2315 31329245 PMC6647355

[36] Kaplowitz PB Slora EJ Wasserman RC Pedlow SE Herman-Giddens ME. Earlier Onset of Puberty in Girls: Relation to Increased Body Mass index and Race. Pediatrics (2001) 108(2):347–53. 10.1542/peds.108.2.347 11483799

[37] Rosenfield RL Lipton RB Drum ML. Thelarche, Pubarche, and Menarche Attainment in Children with normal and Elevated Body Mass index. Pediatrics (2009) 123(1):84–8. 10.1542/peds.2008-0146 19117864

[38] Reinehr T Bosse C Lass N Rothermel J Knop C Roth CL. Effect of Weight Loss on Puberty Onset in Overweight Children. J Pediatr (2017) 184:143–50. 10.1016/j.jpeds.2017.01.066 28238482

[39] Wang Y. Is Obesity Associated with Early Sexual Maturation? A Comparison of the Association in American Boys versus Girls. Pediatrics (2002) 110(5):903–10. 10.1542/peds.110.5.903 12415028

[40] Juul A Magnusdottir S Scheike T Prytz S Skakkebaek NE. Age at Voice Break in Danish Boys: Effects of Pre-pubertal Body Mass index and Secular Trend. Int J Androl (2007) 30(6):537–42. 10.1111/j.1365-2605.2007.00751.x 17459124

[41] Chen R Mick GJ Xu R Zheng D Fan Y Lin X Effect of central Antileptin Antibody on the Onset of Female Rat Puberty. Int J Pediatr Endocrinol (2009) 2009:194807. 10.1155/2009/194807 19946402 PMC2777280

[42] Huang A Roth CL. The Link between Obesity and Puberty: what Is New? Curr Opin Pediatr (2021) 33(4):449–57. 10.1097/MOP.0000000000001035 34173790

[43] Rutters F Nieuwenhuizen AG Verhoef SP Lemmens SG Vogels N Westerterp-Plantenga MS. The Relationship between Leptin, Gonadotropic Hormones, and Body Composition during Puberty in a Dutch Children Cohort. Eur J Endocrinol (2009) 160(6):973–8. 10.1530/EJE-08-0762 19332528

[44] Mancini A Currò D Cipolla C Barini A Bruno C Vergani E Evaluation of Kisspeptin Levels in Prepubertal Obese and Overweight Children: Sexual Dimorphism and Modulation of Antioxidant Levels. Eur Rev Med Pharmacol Sci (2021) 25(2):941–9. 10.26355/eurrev_202101_24663 33577049

[45] Semaan SJ Kauffman AS. Developmental Sex Differences in the Peri-Pubertal Pattern of Hypothalamic Reproductive Gene Expression, Including Kiss1 and Tac2, May Contribute to Sex Differences in Puberty Onset. Mol Cel Endocrinol (2022) 551:111654. 10.1016/j.mce.2022.111654 PMC9889105 35469849

[46] Lee EB Dilower I Marsh CA Wolfe MW Masumi S Upadhyaya S Sexual Dimorphism in Kisspeptin Signaling. Cells (2022) 11(7):1146. 10.3390/cells11071146 35406710 PMC8997554

