# Supplementary material for: Associations of Obesity With Growth and Puberty in Children: A Cross-Sectional Study in Fuzhou, China
Source: Int J Public Health. 2023 May 15;68:1605433. doi: 10.3389/ijph.2023.1605433 (PMC10225596; doi:10.3389/ijph.2023.1605433)
Supplement: Supplementary file 1 [file DataSheet1.docx]

Eligible participants with consent statements

(n=31589)

Exclude:

Did not participate in physical examination (n=4424)

Participants in the physical examination

(n=27165)

Exclude:

1. Missing(n=101)
2. Extreme and invalid data for age and key physical parameters including height and weight(n=185)

The final analyses:

Participants(n=26879)

Boys(n=14647)

Girls(n=12232)

Figure 1S Flow chart of children included in the cross-sectional study.

Table1S Body mass index cutoff values for obesity

| Age (years) | BMI cut-off value for obesity | |
| --- | --- | --- |
|  | Boys | Girls |
| 3 | 18.1 | 18.3 |
| 3.5 | 17.9 | 18.2 |
| 4 | 17.8 | 18.1 |
| 4.5 | 17.8 | 18.1 |
| 5 | 17.9 | 18.2 |
| 5.5 | 18.1 | 18.3 |
| 6 | 18.4 | 18.4 |
| 6.5 | 18.8 | 18.6 |
| 7 | 19.2 | 18.8 |
| 7.5 | 19.6 | 19.1 |
| 8 | 20.1 | 19.5 |
| 8.5 | 20.6 | 19.9 |
| 9 | 21.1 | 20.4 |
| 9.5 | 21.7 | 20.9 |
| 10 | 22.2 | 21.5 |
| 10.5 | 22.7 | 22.1 |
| 11 | 23.2 | 22.7 |
| 11.5 | 23.7 | 23.3 |
| 12 | 24.2 | 23.9 |
| 12.5 | 24.6 | 24.4 |
| 13 | 25.1 | 25.0 |
| 13.5 | 25.5 | 25.5 |
| 14 | 25.8 | 25.9 |
| 14.5 | 26.2 | 26.3 |
| 15 | 26.5 | 26.7 |
| 15.5 | 26.8 | 27.0 |
| 16 | 27.0 | 27.2 |
| 16.5 | 27.3 | 27.4 |
| 17 | 27.5 | 27.6 |
| 17.5 | 27.8 | 27.8 |
| 18 | 28.0 | 28.0 |

The 3-year-old children included all children aged 3.00 to 3.49 years, and the 17.5-year-old children included those aged 17.50 to 18.00 years.

Table 2S Age-specific height differences between boys with obesity and non-obesity

| Age  (years) | Obesity | | Non-obesity | | t value | P value |
| --- | --- | --- | --- | --- | --- | --- |
|  | Height(cm) | n | Height(cm) | n |  |  |
| 3~ | 102.18 ± 3.03 | 6 | 99.29 ± 4.38 | 113 | 1.592 | 0.114 |
| 3.5~ | 103.40 ± 8.48 | 18 | 102.98 ± 3.86 | 277 | 0.557 | 0.578 |
| 4~ | 110.07 ± 6.75 | 36 | 105.75 ± 3.97 | 396 | 7.164 | <0.001 |
| 4.5~ | 112.05 ± 4.04 | 44 | 109.06 ± 4.33 | 459 | 4.411 | <0.001 |
| 5~ | 116.41 ± 3.58 | 44 | 112.41 ± 4.27 | 449 | 5.994 | <0.001 |
| 5.5~ | 119.39 ± 5.15 | 66 | 115.45 ± 4.70 | 419 | 6.253 | <0.001 |
| 6~ | 123.67 ± 5.12 | 70 | 119.11 ± 4.96 | 578 | 7.241 | <0.001 |
| 6.5~ | 126.53 ± 4.48 | 107 | 122.11 ± 4.80 | 726 | 8.952 | <0.001 |
| 7~ | 129.85 ± 4.46 | 124 | 124.91 ± 5.02 | 561 | 10.107 | <0.001 |
| 7.5~ | 131.16 ± 4.67 | 88 | 127.64 ± 5.47 | 515 | 5.687 | <0.001 |
| 8~ | 135.85 ± 6.11 | 112 | 130.49 ± 5.51 | 510 | 9.140 | <0.001 |
| 8.5~ | 137.47 ± 5.78 | 114 | 133.47 ± 5.30 | 532 | 7.187 | <0.001 |
| 9~ | 141.23 ± 5.86 | 96 | 135.89 ± 5.78 | 502 | 8.279 | <0.001 |
| 9.5~ | 144.94 ± 5.89 | 113 | 138.50 ± 5.92 | 456 | 10.365 | <0.001 |
| 10~ | 146.02 ± 6.63 | 79 | 140.98 ± 6.08 | 526 | 6.791 | <0.001 |
| 10.5~ | 148.66 ± 6.25 | 98 | 144.31 ± 6.67 | 553 | 6.014 | <0.001 |
| 11~ | 150.63 ± 6.45 | 74 | 146.78 ± 7.27 | 452 | 4.293 | <0.001 |
| 11.5~ | 155.24 ± 6.69 | 62 | 150.42 ± 8.07 | 405 | 4.472 | <0.001 |
| 12~ | 157.64 ± 7.53 | 44 | 154.74 ± 7.91 | 347 | 2.300 | 0.022 |
| 12.5~ | 163.37 ± 6.39 | 58 | 158.14 ± 8.56 | 464 | 4.494 | <0.001 |
| 13~ | 165.78 ± 7.54 | 66 | 161.85 ± 8.12 | 555 | 3.746 | <0.001 |
| 13.5~ | 168.39 ± 8.08 | 61 | 164.88 ± 7.31 | 553 | 3.514 | <0.001 |
| 14~ | 169.59 ± 5.86 | 61 | 167.18 ± 7.34 | 495 | 2.461 | 0.014 |
| 14.5~ | 171.60 ± 4.76 | 38 | 168.33 ± 6.66 | 325 | 2.934 | 0.004 |
| 15~ | 172.78 ± 7.11 | 54 | 169.36 ± 5.98 | 271 | 3.711 | <0.001 |
| 15.5~ | 172.42 ± 6.92 | 41 | 170.76 ± 6.04 | 327 | 1.634 | 0.103 |
| 16~ | 171.51 ± 6.00 | 31 | 171.60 ± 6.06 | 319 | 0.081 | 0.936 |
| 16.5~ | 172.42 ± 6.12 | 36 | 172.30 ± 6.13 | 329 | 0.110 | 0.913 |
| 17~ | 171.97 ± 6.82 | 25 | 171.81 ± 6.06 | 197 | 0.123 | 0.902 |
| 17.5~18 | 175.26 ± 5.60 | 16 | 172.47 ± 6.49 | 154 | 1.658 | 0.099 |

Table 3S Age-Specific height differences between girls with obesity and non-obesity

| Age  (years) | Obesity | | Non-obesity | | t value | P value |
| --- | --- | --- | --- | --- | --- | --- |
|  | Height(cm) | n | Height(cm) | n |  |  |
| 3~ | 100.75 ± 0.78 | 2 | 98.15 ± 3.38 | 107 | 1.082 | 0.282 |
| 3.5~ | 101.64 ± 5.06 | 7 | 101.54 ± 3.91 | 278 | 0.067 | 0.947 |
| 4~ | 106.49 ± 2.86 | 19 | 104.27 ± 4.24 | 384 | 2.263 | 0.024 |
| 4.5~ | 110.69 ± 3.98 | 22 | 107.83 ± 4.06 | 406 | 3.216 | 0.001 |
| 5~ | 114.63 ± 5.88 | 21 | 111.55 ± 4.65 | 390 | 2.922 | 0.003 |
| 5.5~ | 118.83 ± 3.64 | 25 | 114.90 ± 4.55 | 399 | 4.235 | <0.001 |
| 6~ | 121.36 ± 5.90 | 36 | 118.55 ± 4.65 | 494 | 3.436 | <0.001 |
| 6.5~ | 125.64 ± 5.86 | 50 | 121.09 ± 4.62 | 654 | 6.583 | <0.001 |
| 7~ | 128.11 ± 5.49 | 66 | 123.70 ± 5.00 | 553 | 6.708 | <0.001 |
| 7.5~ | 130.93 ± 5.82 | 64 | 126.73 ± 5.16 | 466 | 6.010 | <0.001 |
| 8~ | 133.98 ± 6.75 | 62 | 129.43 ± 5.73 | 440 | 5.714 | <0.001 |
| 8.5~ | 136.08 ± 6.02 | 59 | 131.95 ± 5.66 | 476 | 5.252 | <0.001 |
| 9~ | 139.68 ± 5.32 | 47 | 135.33 ± 6.21 | 460 | 4.629 | <0.001 |
| 9.5~ | 143.29 ± 5.78 | 38 | 138.78 ± 6.09 | 417 | 4.393 | <0.001 |
| 10~ | 146.45 ± 7.05 | 42 | 141.85 ± 6.43 | 467 | 4.410 | <0.001 |
| 10.5~ | 150.87 ± 6.24 | 45 | 145.65 ± 6.78 | 429 | 4.950 | <0.001 |
| 11~ | 153.84 ± 6.25 | 38 | 148.10 ± 6.75 | 385 | 5.035 | <0.001 |
| 11.5~ | 154.94 ± 6.04 | 38 | 151.75 ± 6.67 | 286 | 2.792 | 0.006 |
| 12~ | 157.23 ± 5.89 | 30 | 154.33 ± 6.17 | 303 | 2.470 | 0.014 |
| 12.5~ | 155.62 ± 6.57 | 31 | 156.54 ± 5.74 | 380 | 0.847 | 0.397 |
| 13~ | 159.07 ± 5.16 | 42 | 157.42 ± 5.49 | 456 | 1.873 | 0.062 |
| 13.5~ | 160.17 ± 5.74 | 21 | 158.78 ± 5.61 | 456 | 1.106 | 0.269 |
| 14~ | 161.65 ± 5.64 | 33 | 158.88 ± 5.63 | 455 | 2.730 | 0.007 |
| 14.5~ | 159.89 ± 5.79 | 18 | 158.91 ± 5.37 | 260 | 0.743 | 0.458 |
| 15~ | 161.80 ± 5.15 | 11 | 160.00 ± 5.69 | 257 | 1.030 | 0.304 |
| 15.5~ | 160.33 ± 6.91 | 15 | 159.89 ± 5.23 | 339 | 0.316 | 0.752 |
| 16~ | 159.18 ± 4.74 | 19 | 159.83 ± 5.04 | 330 | 0.545 | 0.586 |
| 16.5~ | 159.14 ± 6.11 | 9 | 160.65 ± 5.45 | 320 | 0.816 | 0.415 |
| 17~ | 158.72 ± 3.55 | 4 | 159.49 ± 5.56 | 173 | 0.272 | 0.786 |
| 17.5~18 | 160.55 ± 2.90 | 4 | 160.61 ± 5.29 | 94 | 0.021 | 0.984 |

Table 4S Prevalence of testicle development at different ages between boys with obesity and non-obesity

| Age  (years) | Obesity | | Non-obesity | |
| --- | --- | --- | --- | --- |
|  | N  (a*/total) | Prevalence | N  (a*/total) | Prevalence |
| 3~ | 0/5 | 0.00% | 0/113 | 0.00% |
| 3.5~ | 0/18 | 0.00% | 0/275 | 0.00% |
| 4~ | 0/36 | 0.00% | 0/395 | 0.00% |
| 4.5~ | 0/44 | 0.00% | 1/458 | 0.22% |
| 5~ | 0/44 | 0.00% | 0/449 | 0.00% |
| 5.5~ | 0/64 | 0.00% | 1/417 | 0.24% |
| 6~ | 0/70 | 0.00% | 1/578 | 0.17% |
| 6.5~ | 2/107 | 1.87% | 2/724 | 0.28% |
| 7~ | 3/124 | 2.42% | 4/559 | 0.72% |
| 7.5~ | 2/86 | 2.33% | 4/513 | 0.78% |
| 8~ | 9/111 | 8.11% | 6/510 | 1.18% |
| 8.5~ | 9/113 | 7.96% | 27/531 | 5.08% |
| 9~ | 11/95 | 11.58% | 41/502 | 8.17% |
| 9.5~ | 28/111 | 25.23% | 63/456 | 13.82% |
| 10~ | 32/79 | 40.51% | 153/524 | 29.20% |
| 10.5~ | 49/98 | 50.00% | 353/551 | 45.92% |
| 11~ | 60/74 | 81.08% | 306/451 | 67.85% |
| 11.5~ | 55/62 | 88.71% | 341/405 | 84.20% |
| 12~ | 42/44 | 95.45% | 322/347 | 92.80% |
| 12.5~ | 57/58 | 98.28% | 446/463 | 96.33% |
| 13~ | 63/66 | 95.45% | 552/555 | 99.46% |
| 13.5~ | 61/61 | 100.00% | 548/552 | 99.28% |
| 14~ | 61/61 | 100.00% | 494/495 | 98.00% |
| 14.5~ | 38/38 | 100.00% | 325/325 | 100.00% |
| 15~ | 54/54 | 100.00% | 271/271 | 100.00% |
| 15.5~ | 41/41 | 100.00% | 327/327 | 100.00% |
| 16~ | 31/31 | 100.00% | 319/319 | 100.00% |
| 16.5~ | 36/36 | 100.00% | 328/329 | 99.70% |
| 17~ | 25/25 | 100.00% | 197/197 | 100.00% |
| 17.5~18 | 16/16 | 100.00% | 154/154 | 100.00% |

a*, the number of testicle development at Tanner stage 2 or greater at different ages in boys.

n(total)=14617, the data of testicle development in 30 boys is missed.

Table 5S Prevalence of breast development at Tanner stage 2 or greater at different ages between girls with obesity and non-obesity

| Age  (years) | Obesity | | Non-obesity | |
| --- | --- | --- | --- | --- |
|  | N  (a*/total) | Prevalence | N  (a*/total) | Prevalence |
| 3~ | 0/2 | 0.00% | 0/107 | 0.00% |
| 3.5~ | 0/7 | 0.00% | 0/278 | 0.00% |
| 4~ | 0/19 | 0.00% | 4/384 | 1.04% |
| 4.5~ | 0/22 | 0.00% | 4/406 | 0.99% |
| 5~ | 1/21 | 4.76% | 2/390 | 0.51% |
| 5.5~ | 2/25 | 8.00% | 4/399 | 1.00% |
| 6~ | 1/36 | 2.78% | 4/494 | 0.81% |
| 6.5~ | 2/50 | 4.00% | 6/654 | 0.92% |
| 7~ | 3/66 | 4.55% | 17/553 | 3.07% |
| 7.5~ | 11/64 | 17.19% | 15/466 | 3.22% |
| 8~ | 20/62 | 32.26% | 70/440 | 15.91% |
| 8.5~ | 32/59 | 54.24% | 131/476 | 27.52% |
| 9~ | 32/47 | 68.09% | 193/460 | 41.96% |
| 9.5~ | 30/38 | 78.95% | 254/417 | 60.91% |
| 10~ | 38/42 | 90.48% | 363/467 | 77.73% |
| 10.5~ | 45/45 | 100.00% | 379/429 | 88.44% |
| 11~ | 37/38 | 97.37% | 364/385 | 94.55% |
| 11.5~ | 37/38 | 97.37% | 276/286 | 96.50% |
| 12~ | 29/30 | 96.67% | 298/303 | 98.35% |
| 12.5~ | 31/31 | 100.00% | 378/380 | 99.47% |
| 13~ | 42/42 | 100.00% | 454/456 | 99.56% |
| 13.5~ | 21/21 | 100.00% | 456/456 | 100.00% |
| 14~ | 33/33 | 100.00% | 454/455 | 99.78% |
| 14.5~ | 18/18 | 100.00% | 259/260 | 99.62% |
| 15~ | 11/11 | 100.00% | 257/257 | 100.00% |
| 15.5~ | 15/15 | 100.00% | 339/339 | 100.00% |
| 16~ | 19/19 | 100.00% | 330/330 | 100.00% |
| 16.5~ | 9/9 | 100.00% | 320/320 | 100.00% |
| 17~ | 4/4 | 100.00% | 173/173 | 100.00% |
| 17.5~18 | 4/4 | 100.00% | 94/94 | 100.00% |

a*, the number of breast development at Tanner stage 2 or greater at different ages in girls

Table 6S Prevalence of pubic development at different ages between boys with obesity and non-obesity

| Age  (years) | Obesity | | Non-obesity | |
| --- | --- | --- | --- | --- |
|  | N  (a*/total) | Prevalence | N  (a*/total) | Prevalence |
| 3~ | 0/6 | 0.00% | 1/113 | 0.85% |
| 3.5~ | 0/18 | 0.00% | 0/277 | 0.00% |
| 4~ | 0/36 | 0.00% | 0/396 | 0.00% |
| 4.5~ | 0/44 | 0.00% | 1/459 | 0.22% |
| 5~ | 0/44 | 0.00% | 1/449 | 0.22% |
| 5.5~ | 0/66 | 0.00% | 2/419 | 0.48% |
| 6~ | 1/70 | 1.43% | 2/578 | 0.35% |
| 6.5~ | 0/107 | 0.00% | 3/726 | 0.41% |
| 7~ | 0/124 | 0.00% | 0/561 | 0.00% |
| 7.5~ | 1/88 | 1.14% | 1/515 | 0.19% |
| 8~ | 0/112 | 0.00% | 6/510 | 1.18% |
| 8.5~ | 0/114 | 0.00% | 4/532 | 0.75% |
| 9~ | 0/96 | 0.00% | 1/502 | 0.20% |
| 9.5~ | 2/113 | 1.77% | 6/456 | 1.32% |
| 10~ | 1/79 | 1.27% | 13/526 | 2.47% |
| 10.5~ | 7/98 | 7.14% | 36/553 | 6.51% |
| 11~ | 17/74 | 22.97% | 83/452 | 18.36% |
| 11.5~ | 19/62 | 30.65% | 142/405 | 35.06% |
| 12~ | 23/44 | 52.27% | 187/347 | 53.89% |
| 12.5~ | 47/58 | 81.03% | 346/464 | 74.57% |
| 13~ | 59/66 | 89.39% | 464/555 | 83.60% |
| 13.5~ | 56/61 | 91.80% | 513/553 | 92.77% |
| 14~ | 59/61 | 96.72% | 473/495 | 95.56% |
| 14.5~ | 38/38 | 100.00% | 321/325 | 98.77% |
| 15~ | 54/54 | 100.00% | 269/271 | 99.26% |
| 15.5~ | 41/41 | 100.00% | 327/327 | 100.00% |
| 16~ | 31/31 | 100.00% | 318/319 | 99.69% |
| 16.5~ | 36/36 | 100.00% | 328/329 | 99.70% |
| 17~ | 25/25 | 100.00% | 196/197 | 99.49% |
| 17.5~18 | 16/16 | 100.00% | 154/154 | 100.00% |

a*, the number of testicle development at Tanner stage 2 or greater at different ages in boys

Table 7S Prevalence of pubic development at Tanner stage 2 or greater at different ages between girls with obesity and non-obesity

| Age  (years) | Obesity | | Non-obesity | |
| --- | --- | --- | --- | --- |
|  | N  (a*/total) | Prevalence | N  (a*/total) | Prevalence |
| 3~ | 0/2 | 0.00% | 0/107 | 0.00% |
| 3.5~ | 0/7 | 0.00% | 0/278 | 0.00% |
| 4~ | 0/19 | 0.00% | 1/384 | 0.26% |
| 4.5~ | 0/22 | 0.00% | 0/406 | 0.00% |
| 5~ | 0/21 | 0.00% | 0/390 | 0.00% |
| 5.5~ | 0/25 | 0.00% | 2/399 | 0.50% |
| 6~ | 0/36 | 0.00% | 0/494 | 0.00% |
| 6.5~ | 0/50 | 0.00% | 2/654 | 0.31% |
| 7~ | 0/66 | 0.00% | 0/553 | 0.00% |
| 7.5~ | 1/64 | 1.56% | 5/466 | 1.07% |
| 8~ | 1/62 | 1.61% | 7/440 | 1.59% |
| 8.5~ | 3/59 | 5.08% | 4/476 | 0.84% |
| 9~ | 2/47 | 4.26% | 13/460 | 2.83% |
| 9.5~ | 5/38 | 13.16% | 39/417 | 9.35% |
| 10~ | 18/42 | 42.86% | 85/467 | 18.20% |
| 10.5~ | 21/45 | 46.67% | 137/429 | 31.93% |
| 11~ | 30/38 | 78.95% | 209/385 | 54.29% |
| 11.5~ | 33/38 | 86.84% | 216/286 | 75.52% |
| 12~ | 29/30 | 96.67% | 266/303 | 87.79% |
| 12.5~ | 29/31 | 93.55% | 368/380 | 96.84% |
| 13~ | 42/42 | 100.00% | 450/456 | 98.68% |
| 13.5~ | 21/21 | 100.00% | 450/456 | 98.68% |
| 14~ | 33/33 | 100.00% | 453/455 | 99.56% |
| 14.5~ | 18/18 | 100.00% | 259/260 | 99.62% |
| 15~ | 11/11 | 100.00% | 257/257 | 100.00% |
| 15.5~ | 15/15 | 100.00% | 339/339 | 100.00% |
| 16~ | 19/19 | 100.00% | 329/330 | 99.70% |
| 16.5~ | 9/9 | 100.00% | 320/320 | 100.00% |
| 17~ | 4/4 | 100.00% | 173/173 | 100.00% |
| 17.5~18 | 4/4 | 100.00% | 94/94 | 100.00% |

a*, the number of breast development at Tanner stage 2 or greater at different ages in girls.

Table 8S. The relationship between age at menarche time and obesity in girls

| Group | Menarche time(years) | | | | | | | | | Chi-square | | *p* value | |
| --- | --- | --- | --- | --- | --- | --- | --- | --- | --- | --- | --- | --- | --- |
|  | <10 | 10~11 | 11~12 | 12~13 | 13~14 | 14~15 | >15 | total |  | |  | |  |
| Obesity | 9 (3.25%) | 64 (23.10%) | 95 (34.30%) | 78 (28.16%) | 26 (9.39%) | 5 (1.81%) | 0 (0.00%) | 277 (100%) | 45.756 | | <0.001 | |  |
| Non-obesity | 43 (1.14%) | 452 (11.98%) | 1252 (33.19%) | 1323 (35.07%) | 576 (15.27%) | 117 (3.10%) | 9 (0.24%) | 3772  (100%) |  |  |  |  |  |
